# Fabrication of In Vitro Cancer Microtissue Array on Fibroblast-Layered Nanofibrous Membrane by Inkjet Printing

**DOI:** 10.3390/ijms18112348

**Published:** 2017-11-07

**Authors:** Tae-Min Park, Donggu Kang, Ilho Jang, Won-Soo Yun, Jin-Hyung Shim, Young Hun Jeong, Jong-Young Kwak, Sik Yoon, Songwan Jin

**Affiliations:** 1 Research Institute, Femtobiomed Co., Ltd., 700, Pangyo-ro, Seongnam-si, Gyeonggi-do 13516, Korea; taemin333@gmail.com; 2 Department of Mechanical System Engineering, Korea Polytechnic Univsersity, 237 Sangidaehak-ro, Siheung-si, Gyoenggi-do 15073, Korea; kdgplant@naver.com; 3 Department of Advanced Convergence Technology, Korea Polytechnic Univsersity, 237 Sangidaehak-ro, Siheung-si, Gyoenggi-do 15073, Korea; bluenadsky@gmail.com; 4 Research Institute, T&R Biofab Co., Ltd., 237 Sangidaehak-ro, Siheung-si, Gyoenggi-do 15073, Korea; wsyun@tnrbiofab.com; 5 Department of Mechanical Engineering, Korea Polytechnic Univsersity, 237 Sangidaehak-ro, Siheung-si, Gyoenggi-do 15073, Korea; happyshim@kpu.ac.kr; 6 School of Mechanical Engineering, Kyungpook National University, 80 Daehak-ro, Buk-gu, Daegu 702-701, Korea; yhjeong@knu.ac.kr; 7 Department of Pharmacology, Ajou University School of Medicine, Suwon 442-721, Korea; jykwak@ajou.ac.kr; 8 Department of Anatomy, Pusan National University School of Medicine, Yangsan 626-770, Korea; sikyoon@pusan.ac.kr

**Keywords:** in vitro cancer model, inkjet cell printing, microtissue array, cancer drug discovery, electrospinning, nanofibrous membrane

## Abstract

In general, a drug candidate is evaluated using 2D-cultured cancer cells followed by an animal model. Despite successful preclinical testing, however, most drugs that enter human clinical trials fail. The high failure rates are mainly caused by incompatibility between the responses of the current models and humans. Here, we fabricated a cancer microtissue array in a multi-well format that exhibits heterogeneous and batch-to-batch structure by continuous deposition of collagen-suspended Hela cells on a fibroblast-layered nanofibrous membrane via inkjet printing. Expression of both Matrix Metalloproteinase 2 (MMP2) and Matrix Metalloproteinase 9 (MMP9) was higher in cancer microtissues than in fibroblast-free microtissues. The fabricated microtissues were treated with an anticancer drug, and high drug resistance to doxorubicin occurred in cancer microtissues but not in fibroblast-free microtissues. These results introduce an inkjet printing fabrication method for cancer microtissue arrays, which can be used for various applications such as early drug screening and gradual 3D cancer studies.

## 1. Introduction

Animal models are limited in their ability to mimic extremely complex carcinogenic and physiological processes in humans and more than 90% of drugs that pass animal trials fail in the clinical trials [1,2]. Various researchers have developed biomimetic models that can produce results similar to actual responses in humans, and three-dimensionally (3D) cultured cells have been found to better represent human tissue than two-dimensionally (2D) cultured cells [3,4,5,6,7,8,9]. Most cells in tissues are surrounded by an extracellular matrix (ECM), which provides a structural chemical environment for cells and interacts with cells/ECM to regulate diverse functions including proliferation, differentiation, and migration [10]. Therefore, we employed an electrospun nanofibrous membrane (NF) whose structure is similar to that of the extracellular matrix. The cell-laden NF has various morphologies, in contrast to two-dimensionally cultured cells, and the cells interact more actively based on the surface characteristics of this membrane.

**Figure 1:**
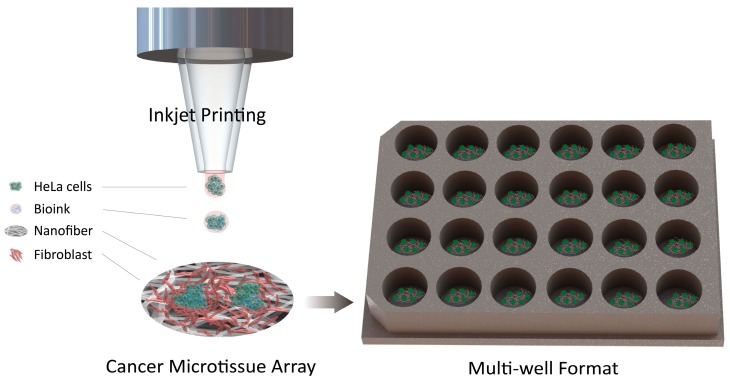
Schematic illustration of cancer microtissue array.

In the drug discovery process, multiple tests are required to verify various factors such as efficacy, safety, and dose of the drug [11]. Current 3D culture models have various difficulties such as limited control over cell positioning and multiply arrayed cells and batch-to-batch variability. To this end, this study developed and evaluated a patterned cancer microtissue array with controlled size and shape as well as batch-to-batch structures using an inkjet printer. Multiple tests can be simultaneously performed on the developed microtissue array (Figure 1). The inkjet printer enabled precise adjustment of the spatial placement of the tissue formed on the NF with microscale resolution, and quantitative cell seeding was possible, thus improving the repeatability and reproducibility of the model.

The tumor microenvironment is constantly evolving owing to tissue remodeling and changes in the recruitment of stromal cells including a diversity of immune cells with angiogenesis. As the tumor becomes larger, the phenotype of cancer cells changes to aggressive, and in the tumor microenvironment, the extracellular matrix also changes [12]. In addition, fibroblasts, which are cancer stromal cells, play an important role in tumor progression, growth, and metastasis [13]. Therefore, developing functional and gradational cancer models that mimic the tumor microenvironment, including stromal cells, are required for testing drugs and investigating mechanisms of tumor progression as well as cancer metastasis.

This study used cervical cancer cells, which are invasive and aggressive and closely associated with matrix metalloproteinases (MMPs). In particular, MMP2 and MMP9 have been found to be the most important in the degradation of the basement membrane and extracellular matrix needed for cells to acquire invasive capabilities [14]. In this study, MMP2 and MMP9 levels were measured through enzyme-linked immunosorbent assay (ELISA) to verify the effect of co-culturing with fibroblasts. The 3D structure of the fabricated cancer microtissue was examined using histological analysis, scanning electron microscopy (SEM), and confocal microscopy by staining for human papillomavirus 18 (HPV18), which is specifically found in cervical cancer cells. Finally, we treated the model with an anticancer drug for assessment of the drug response.

## 2. Results

### 2.1. Concept of the Cancer Microtissue Array

#### 2.1.1. Electrospinning and Nanofibrous Membrane

**Figure 2:**
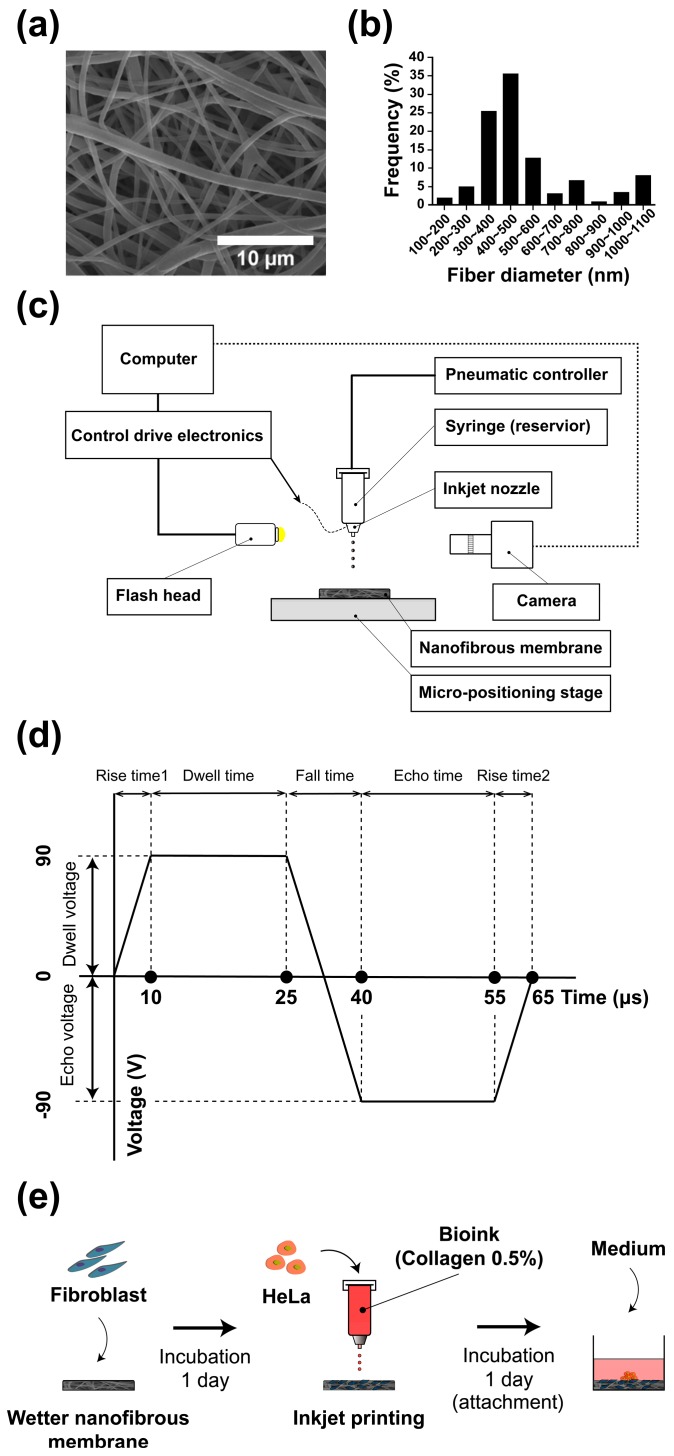
Scanning electron microscopy (SEM) image of the electrospun nanofibrous membrane (NF) (**a**); diameter distribution of the nanofibers (**b**); schematic representation of the inkjet printer (**c**); bipolar waveform for piezo actuation (**d**); and schematic representation of the cancer microtissue fabrication process (**e**).

The diameter distribution of the nanofibers characterized by SEM imaging (Figure 2a) is shown in Figure 2b; the nanofiber diameter ranged from 150 to 1200 nm, and the peak frequency of the diameter was 350 to 500 nm.

#### 2.1.2. Inkjet Printer Setup

Figure 2c presents a schematic representation of the inkjet printer setup. The piezoelectric drop-on-demand print-head had an inner diameter of 80 μm (MJ-AL-01-80-8MX, Microfab, Inc., Plano, TX, USA), and an electronic control drive (CT-M3-04, Microfab, Inc.) and a pressure controller (Super EX-V7, Musashi Engineering, Inc., Tokyo, Japan) were coupled. The pressure controller applied negative pressure to prevent leakage of bioink from the nozzle.

The printing droplet frequency was set at 10 Hz, and the distance between the printer head and NF was maintained at 2 mm or less to minimize physical damage to the cells during printing. The piezo actuation of the inkjet printing was as shown in Figure 2d. The droplet pattern could be printed by directly moving the NF using a triaxial microstage.

#### 2.1.3. Schematic Illustration of Cancer Microtissue Array Process

To fabricate cancer microtissue samples, droplets of the 0.5% collagen bioink containing cancer cells were dropped 150 times using an inkjet printer on a NF as shown in Figure 2e. Three different conditions for the NF were tested: the first was bare membrane, and the others were fibroblast-layered NF with different cell seeding densities.

### 2.2. Cell Viability and Diameter

#### 2.2.1. Cell Viability on Glass and Nanofibrous Membrane

**Figure 3:**
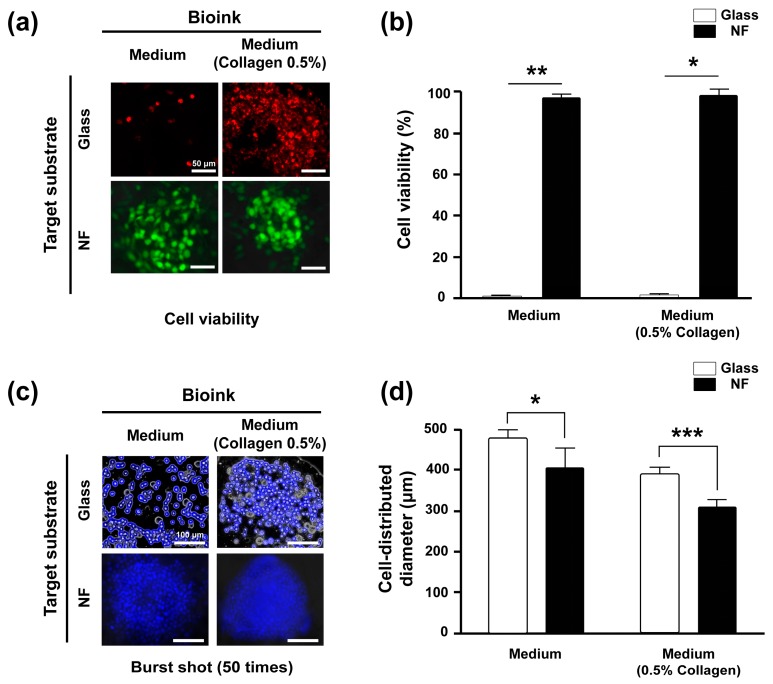
Cell viability and aggregate formation of cancer cells printed on glass and NF; Live/Dead assays of HeLa cells printed on the NF and glass. Cells were suspended in culture medium and culture medium containing 0.5% collagen. The live and dead cells are denoted in green and red, respectively (**a**). Viability of the printed cancer cells (**b**). Shapes of droplets printed on glass and NF (**c**); white arrows show protruding cells. Cells were suspended in culture medium and culture medium containing 0.5% collagen. Cell-distributed diameter according to the number of printed droplets (burst shot; 50 times) on glass and the NF (**d**). All experimental samples were *n* > 3. * *p* < 0.01, ** *p* < 0.005, *** *p* < 0.001. Unmarked scale bars in Figure 3a and 3c, 50 μm and 100 μm, respectively.

In this study, we aimed to pattern four cancer microtissue arrays (diameter of each cancer microtissue, approximately 500 μm) within a diameter of 10 mm. In order to insure the consistency in size of each microtissue, the same number of cells needs to be seeded at one point in designed positions. Inkjet cell printing technology was used for microtissue array fabrication since it enables high resolution patterning of specific cells with soluble biomaterials, providing an ideal environment for fabricating the biomimetic samples. The viability of printed cells compared with that of cells suspended only in medium (bioink) or medium containing 0.5% collagen (collagen-containing bioink) was evaluated through a Live/Dead assay (Figure 3a,b). The cell viability on the NF was approximately 100%, whereas that on glass was approximately 0% owing to drying out of bioink, as shown in Figure 3c. Therefore, it was confirmed that the NF provided a sufficient niche for cells in terms of a medium-infiltrated substrate, owing to its large surface-to-volume ratio.

#### 2.2.2. Cell Aggregate Formation on Glass and NF

As shown in Figure 3c, the droplet of collagen-containing bioink printed on the glass had smaller diameter and a narrower cell distribution area than that of the bioink only, indicating that the cancer cells were more strongly distributed at the central area of the droplets. Similarly, the cell-distributed area of collagen-containing bioink on the NF was smaller than that of the bioink. Consequently, the cell-distributed area of collagen-containing bioink on the NF was the narrowest (Figure 3d), owing to the robust structure of randomly intertwined interstitial fibers and viscous collagen. Therefore, printing on a non-porous two-dimensional substrate such as a glass was unsuitable for fabricating cell aggregates, and the NF was a rather suitable alternative. Inkjet printing with collagen-containing bioink on an NF was optimal for fabricating cancer cell aggregates.

### 2.3. Diameter of Cancer Microtissue

**Figure 4:**
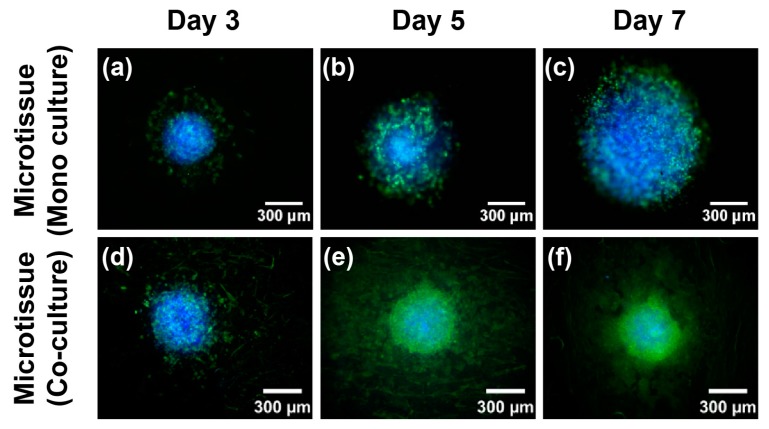

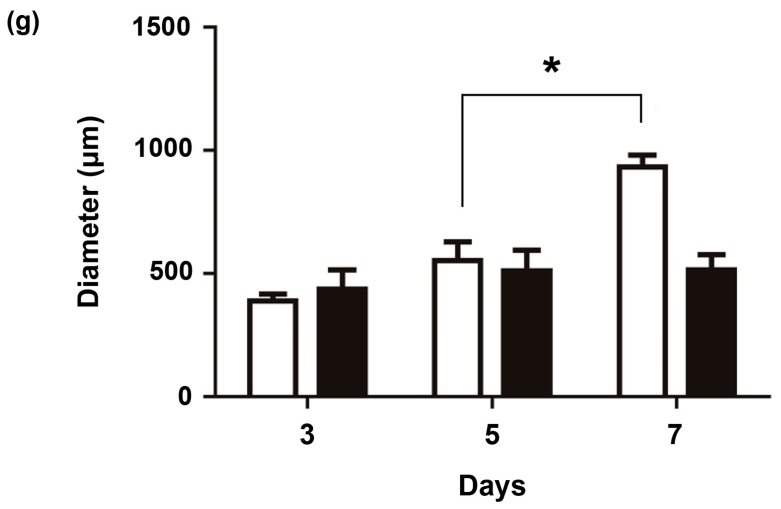
Immunofluorescence staining images of the mono-cultured cancer microtissue (**a**–**c**) and fibroblast-layered microtissue (**d**–**f**). Change in diameter of cancer microtissues with time (**g**). Blue, cell nucleus; green, F-actin staining. All experimental samples were *n* > 3. (* *p* < 0.01).

We next compared mono-cultured cancer microtissue and co-cultured (i.e., fibroblast-layered NF; $1\times 10^{4}$ cells/mL) cancer microtissue. Figure 4 shows the remodeling and diameter of the printed microtissue over time. On days 3, 5, and 7, the printed microtissue on fibroblast-layered NF (Figure 4d–f) maintained its size better than the mono-cultured microtissue (Figure 4a–c). The diameter of the fibroblast-layered microtissue increased by approximately 14% from day 3 to day 5, but the shape was almost identical on days 5 and 7, as shown in Figure 4g. In contrast, the diameter of the mono-cultured microtissue increased by 30% between day 3 and day 5 and by approximately 40% between day 5 and day 7. Therefore, the fibroblast-layered microtissue is more appropriate as a cancer model because it maintains the cancer-aggregate formation over time and hence does not have temporal constraints in drug screening applications.

### 2.4. Cancer Microtissue Formation on Fibroblast Layer

**Figure 5:**
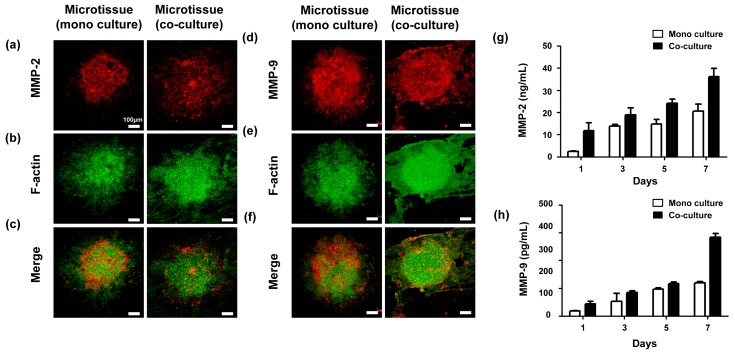
Cancer microtissues (**a**–**f**); monocultured microtissue and fibroblast-layered microtissue (1 × 10^4^ cell/mL) on day 7. Red, MMP2 (**a**) and MMP9 (**d**); green, F-actin (**b**,**e**). MMP2 (**g**) and MMP9 (**h**) expression in the mono-cultured and fibroblast-layered microtissues was quantified using ELISA. All experimental samples were *n* > 3. Unmarked Scale bars, 100 μm.

To verify the effect of the interstitial fibroblast layer on the NF for generation of an in vitro cancer microtissue array, the mono-cultured microtissue and the fibroblast-layered microtissues (1 $\times 10^{4}$ cells/mL) were examined using immunofluorescent staining on day 7 (Figure 5a–f). The cancer cells in monoculture models were spread widely without forming cell aggregates, whereas the fibroblast layer stabilized the forms of the cell aggregates.

The expression of MMP2 and MMP9 was measured by ELISA using the monocultured and fibroblast-layered microtissue (1 $\times 10^{4}$ cells/mL), respectively. As shown in Figure 5g,h, MMP2 and MMP9 were upregulated in the fibroblast-layered microtissue rather than in the mono-cultured microtissue. Especially on day 7, the MMP2 and MMP9 expression of the fibroblast-layered microtissue was approximately 1.5-fold and 2-fold higher than that of the monocultured microtissue. The high levels of MMP2 and MMP9, which are important factors in extracellular matrix remodeling [14], are evidence of good biomimicry, and we can conclude that the fibroblast-layered microtissue better represents cancer tissues than the monocultured microtissue.

### 2.5. 3D Formation and Shape of The Cancer Microtissue

**Figure 6:**
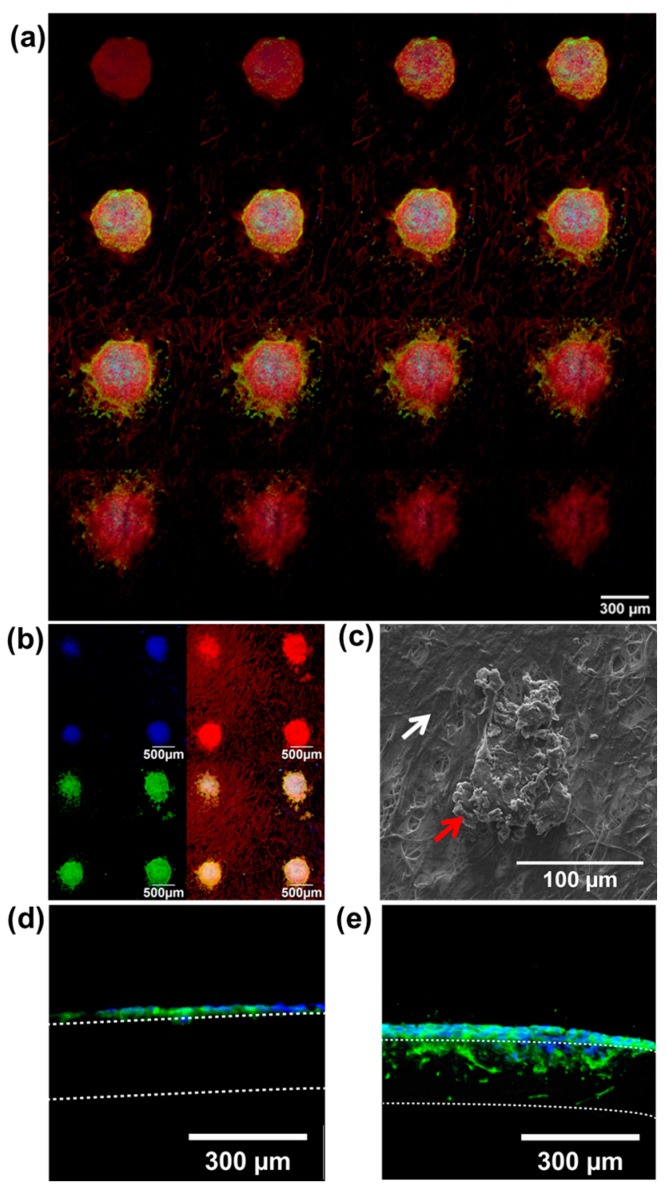
Three-dimensional shapes of cancer microtissues. (**a**,**b**) Confocal microscope images of the cancer microtissues. (**a**) Confocal stacking image (3 μm step; 16 layers) and (**b**) patterned cancer microtissue. (**a**,**b**) Green indicates HPV18, and red indicates F-actin. (**c**) SEM image (white arrow: fibroblast, red arrow: cancer tissue). (**d**,**e**) Frozen section images, (**d**) mono-cultured sample, and (**e**) co-cultured sample. Blue indicates Hoechst 33342 (**d**,**e**); only cancer cells are stained. Green indicates F-actin; both fibroblast and cancer cells are stained. All experimental samples were *n* > 3.

3D characteristics of the fabricated microtissue are shown in Figure 6. Confocal imaging showed that the HeLa cells cultured on the fibroblast-layered NF formed a 3D, disk-like structure with a thickness of approximately 50 μm. Furthermore, fluorescence staining showed that HPV18, which is specifically associated with cervical cancer, was strongly expressed by the patterned cancer tissues, and the cancer tissues were easily identified in the fibroblast-layered microtissue (Figure 6b). As shown in Figure 6b, we were able to prepare four individual cancer microtissues within one well of a 96-well plate, and those tissues were maintained for up to seven days. SEM (Figure 6c) showed that the surrounding fibroblasts (Figure 6c; white arrow) and the formed cancer tissue (Figure 6c; red arrow) were clearly distinguished. This shows that the cancer tissue has a three-dimensional structure, in contrast to the fibroblasts that grow with no layering on the surface of the NF. Histological analysis revealed that the mono-cultured microtissue was thinner than fibroblast-layered microtissue (Figure 6d), whereas the HeLa cells in the fibroblast-layered microtissue were thicker (Figure 6e). Interestingly, some of the HeLa cells penetrated into the NF through the interstitial fibroblast layer (Figure 6 d,e; white dotted lines). In fact, most carcinomas begin in a tissue that lines the inner or outer surfaces with flat and circular structures and then gradually spread and invade into adjacent layers. The fabricated microtissues also adopted flat and circular structures. However, the fibroblast-layered microtissue progressively developed the real tissue-like structure of invasive carcinoma as shown in Figure 6e.

### 2.6. Reaction of the Drug-Treated Cancer Microtissue

**Figure 7:**
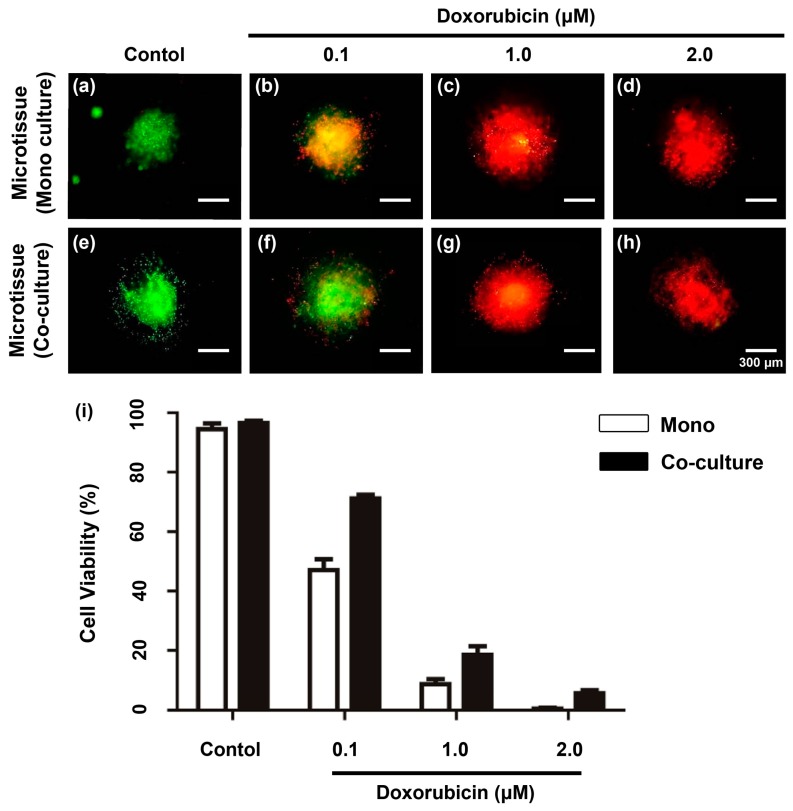
Drug test on day-7 cancer microtissue. (**a**–**h**) Cancer microtissues with Live/Dead staining; (**a**–**d**) mono-cultured sample, (**e**–**h**), co-cultured sample. (**a**,**e**) Standard samples with no doxorubicin treatment. (**b**–**d**,**f**–**h**) Samples with doxorubicin treatment, (**b**,**f**) 0.1, (**c**,**g**) 1.0, and (**d**,**h**) 2.0 μm doxorubicin. (**i**) Graph of viability of cancer micro tissue models after two days of treatment with doxorubicin. All experimental samples were *n* > 3. Unmarked Scale bars, 300 μm.

Doxorubicin (0.1, 1.0, and 2.0 μM) was added to the cancer microtissues at day 7, and the resistance of the cells to the anticancer drug was examined by screening through the Live/Dead staining method (Figure 7a–h). Moreover, the expression of MMP2 and MMP9 was verified through ELISA (Figure 7i). Live/Dead staining two days after doxorubicin treatment revealed that the fibroblast-layered microtissue had higher drug resistance than the mono-cultured microtissue, especially for 0.1 and 1.0 μM doxorubicin (Figure 7b,c,f,g). This result verifies that the fibroblast-layered samples had less reduction of viability (Figure 7i) than mono-cultured microtissue, in which many cancer cells were killed by increasing concentrations of doxorubicin. Thus, the interstitial fibroblast layer conjugated with cancer micro tissue had stronger resistance to anticancer drugs with increasing concentration.

## 3. Discussion

Environmental factors have significant biochemical influences on cells [15]. A two-dimensional culture is advantageous in terms of manipulation to observe cells and extract proteins and mRNA. However, most of these cells are flat, in contrast to their actual shapes in the human body, and in many cases, the experimental results are different from the actual in vivo situations. In contrast, a three-dimensional culture model is more complex and difficult to manipulate and evaluate than a two-dimensional culture model, but it can accurately mimic the 3D structures of cells in the human body, and their behaviors and the expression of proteins and mRNA in actual in vivo situations [16]. Thus, in this study, fibroblasts were cultured with NF to provide a sufficient niche for cancer cells, and the co-cultured microtissue showed a different tissue-like structure and drug resistance compared to the monoculture.

The complex test processes and high throughput of each cell are the greatest obstacles to quantitative and in-depth molecular analysis of single cells or cell populations. The distribution of cells can be quantified by patterning cells to aid in the study of cell processes, such as reactions between individual tissues and cells. The advantage of patterning technology is the exposure of tissues to the same conditions owing to the arrangement of tissues. This study exploited inkjet printing to arrange cancer microtissues on nanofibrous membranes and analyzed the viability, size changes, and other characteristics of the patterned microtissues. The analysis showed that the viability of the cells was close to 100% when they were printed on a nanofibrous membrane using an inkjet printer, and the cancer microtissues produced and patterned using inkjet printing maintained their initial patterns even after culturing for one week. Such patterned cancer microtissues can be multi-screened, thus enabling simultaneous verification of the changes in the size of multiple cancer microtissues and observation of various tissues.

The rapid evaporation of droplets is one of the greatest drawbacks of the inkjet cell printer. The easy evaporation of droplets in micro units makes it difficult to culture cells, and many researchers have addressed this problem by printing hydrogels in wells containing a crosslinking agent [17,18,19]. In contrast, in this study, a nanofibrous membrane soaked in medium was used as the substrate, and printing on this wetted nanofibrous membrane prevented the rapid evaporation of droplets. The nanofibrous membrane provided an appropriate niche for inkjet printing without the use of a crosslinking agent when culturing the printed cells and also provided structural and biochemical elements of the extracellular matrix when culturing the cells together with fibroblasts. We also showed that inkjet printing on nanofibrous membrane is advantageous in terms of shaping and maintaining cell aggregates.

In future studies, the immunity network based on the interaction between immune cells and the fabricated cancer microtissues will be simulated, and drug tests with a sample with such an immunity network are expected to produce results that are closer to the results obtained from the human body. The significance of this study is the simulation of in vitro cancer tissues in drug tests and the production of highly reliable samples. The failure rates of clinical trials are expected to decrease as the reactions to drugs would better replicate those in the human body, and this will have positive effects on pharmaceutical discovery processes with low success rates.

## 4. Materials and Methods

### 4.1. Fabrication of NFs by Electrospinning

NF was fabricated by a previously reported electrospinning method [20,21]. Briefly, nanofibers were produced by diluting high molecular weight (80,000 MW) polycaprolactone (Sigma-Aldrich, St. Louis, MO, USA) at a concentration of 17% in a 1:1 mixture of chloroform (99.5%, Samchun Pure Chemical Co., Ltd., Seoul, Korea) and dimethylformamide (Sigma-Aldrich). During electrospinning, the temperature and humidity were maintained at 20–21 °C and 50–55%, respectively. The voltage, tip-to-collector distance, and flow rate of the solution were maintained at 15 kV, 18 cm, and 0.5 mL/h, respectively. The fabricated NF was dried for 24 h to evaporate the remaining solvent, and the dried membrane was then baked in an oven at 60 °C for 10 s to improve the interconnectivity of interstitial fibers before they were cut to a diameter of 10 mm. The fabricated NF was soaked in 70% ethanol and sterilized under an ultraviolet lamp overnight. Then, the sample was washed out three times with phosphate-buffered saline (PBS; pH 7.4, Gibco, Rockvile, MD, USA) and soaked in Dulbecco’s modified Eagle’s medium (DMEM, GenDEPOT) containing 10% fetal bovine serum (FBS, GenDEPOT) and 1% penicillin-streptomycin (Pen Strep, Gibco). The diameter distribution of the nanofibers characterized by an SEM image (Figure 2a) is shown in Figure 2b; the diameter of the nanofibers was estimated to be 400–500 nm.

### 4.2. Cell and Bioink Preparation

Fibroblasts (CCD-1112SK, ATCC, Manassas, VA, USA) and HPV18-positive cervical cancer cells (HeLa; KCLB, Seoul, Korea) were cultured with a medium containing 10% FBS and 1% Pen Strep in a 100-mm Petri dish (Nunclon Delta Surface, Thermo Fisher Scientific, Waltham, MA, USA). When cell confluency reached 80%, the cells were washed three times with PBS and treated with 2 mL of trypsin-EDTA (0.25%; Gibco) to produce single-cell units before culturing in 5% CO_2_ at 37 °C for 2 min. After culturing, the detached cells were collected in a 15-mL conical tube into which culture medium was added, diluted 10 times to neutralize the trypsin, and centrifuged for 3 min at 1200 rpm. The cells obtained after removal of the supernatant were mixed with the culture medium and then used in the experiment.

The fibroblasts ($1\times 10^{4},\;1\times 10^{5}$ cells/mL) were seeded on NF with a diameter of 10 mm. After culturing in an incubator overnight, fibroblast-layered NF or bare NF was used as a substrate for inkjet cell printing.

For the cell-laden bioink, cell suspensions ($3\times 10^{7}$ cells/mL) were produced containing 0.5% collagen (collagen type I, Thermo Fisher Scientific) or cell suspension alone.

### 4.3. Inkjet Cell Printing

Inkjet printing technology that improves cell aggregation is essential for fabricating cancer microtissues. Therefore, the formation of the printed droplets on different substrates (glass and NF) and bioink was verified. To examine the effects of the bioink on the droplets, the culture medium with or without 0.5% collagen was mixed with HeLa cells ($4\times 10^{7}$ cells/mL) stained with Hoechst 33342 (Thermo Fisher Scientific), and this mixture was then used as the bioink for printing. To examine the effect of the substrate, the droplets were printed on a glass and the NF wetted in the medium.

To fabricate cancer microtissue samples, droplets of the 0.5% collagen bioink containing cancer cells were dropped 150 times using an inkjet printer on a NF as shown in Figure 2e. Three different conditions of NF were tested: the first was bare membrane, and the others ware fibroblast-layered membranes ($1\times 10^{4}$ cells/mL). An array of a maximum of four microtissues was patterned on the membrane (∅10 mm). Immediately afterwards, the microtissues were cultured for 2 h in 5% CO_2_ at 37 °C to enable the cancer cells to positively adhere. A sufficient amount of culture medium was added after adhesion of the cancer cells to the sample. Furthermore, for the co-cultured sample, fibroblasts were seeded on the NF and then soaked in the medium. The cells were then cultured in 5% CO_2_ at 37 °C for one day before the cancer cells were printed as described above (Figure 2d).

### 4.4. Cell Viability

To verify the viability of the printed cells, a Live/Dead kit (Invitrogen) was used in this experiment according to the manufacturer’s recommended procedure. In brief, 20 μL of 2 mM EthD-1 stock solution enclosed in the product was added to 10 mL of PBS and mixed by vortexing. Then, 5 μL of 4 mM calcein AM stock solution was added to the mixed solution, which was vortexed again to produce a live/dead solution. The samples were washed out three times with PBS and treated with the live/dead solution on the NF. Then, the specimen was cultured at room temperature, i.e., 20 °C, for 40 min.

Specifically, to compare the viability of printed cells on dried glass and wetted nanofibrous membrane (Figure 3), the printed cells were gently subjected to the live/dead assay immediately after the printing. Thereafter, the sample was cultured in situ at 25 °C for 30 min.

### 4.5. Immunocytochemistry/Immunofluorescence

The sample was fixed by treatment with 4% paraformaldehyde for 20 min at room temperature and then washed three times with PBS to remove the paraformaldehyde. The washed sample was treated with 0.1% Triton X-100 for 15 min at room temperature to ensure permeability and then washed out with PBS three times. BSA buffer (1%) was used as the blocking buffer, and after treatment for 1 h at room temperature, the sample was washed with PBS three times. Then, the specific antibodies (MMP-2, MMP-9, and HPV18) were diluted in PBS at a ratio of 1:500. The primary and secondary antibodies were applied to the sample for 1 h at room temperature without light exposure.

### 4.6. Enzyme-Linked Immunosorbent Assay (ELISA)

Human MMP2 (ab100606, Abcam, Cambridge, UK) and MMP9 (ab100610, Abcam, Cambridge, UK) ELISA kits were used to measure the level of MMP2 and MMP9. The procedure was followed the manufacturer’s instructions. To summarize, 100 μL of the standard sample, which was the benchmark, and 100 μL of the specific experimental sample to be measured in this experiment were added to the well plates and cultured for 150 min at room temperature. Then, the solution was removed and the plates were washed with distilled water and PBS-Triton. Then, 100 μL of biotinylated (MMP2, MMP9) detection antibody was added to the well plates and mixed carefully for 1 h at room temperature. The solution was removed and the well plates were washed again with distilled water and PBS-Triton. Then, 100 μL of HRP–streptavidin solution was added to each well plate and carefully mixed for 45 min at room temperature. The well plates were washed with distilled water and PBS-Triton again, and 100 μL of TMB one-step substrate reagent was added to each well plate and carefully mixed for 30 min at room temperature without light exposure. Finally, 50 μL of the stop solution was added to each well plate and the absorbance at 450 nm was measured using a microreader. The amount of MMP in the cancer microtissue sample was compared with that in the standard sample.

### 4.7. Drug Testing

Doxorubicin (1 mg, Sigma Aldrich) was used to verify the drug resistance of the fabricated samples. The doxorubicin was diluted to 0.1, 1.0, and 2.0 μM in cell culture medium and added to the day-7 co-cultured samples, after which further culture was performed in 5% CO_2_ at 37 °C. Then, the amounts of MMP2 and MMP9 expressed after one and two days of administration were measured with ELISA, and a Live/Dead assay was used to examine the drug resistance of the fabricated cancer tissues.

### 4.8. Statistical Analysis

All of the data were presented as the mean ± standard deviation (SD). Statistical significance was defined as * *p* < 0.01, ** *p* < 0.005, *** *p* < 0.001.

## 5. Conclusions

This study fabricated a cancer microtissue array by inkjet printing cancer cells on NF on which fibroblasts were cultured. The cancer microtissues fabricated by printing cells on NF with a bioink containing 0.5% collagen showed the highest performance. The shapes of the cell aggregates were maintained well when the printed cancer cells were co-cultured with fibroblasts, and the tissues maintained their shapes and sizes with no significant variations until day 7 after production. The patterned cancer microtissues could be distinguished by staining of HPV18 in the fibroblast-layered microtissue, and their three-dimensional structures were verified using frozen sections, SEM, and confocal microscopy. The fibroblast-layered microtissue showed higher expression of MMP2 and MMP9 compared with the monocultured microtissue and higher resistance to doxorubicin.
